# The effect of prions on cellular metabolism: The metabolic impact of the [RNQ^+^] prion and potential role of native Rnq1p

**DOI:** 10.21203/rs.3.rs-2511186/v1

**Published:** 2023-02-28

**Authors:** Tyler Howell-Bray, Lee Byrne

**Affiliations:** Canterbury Christ Church University

**Keywords:** prion, mass spectrometry, metabolomics, yeast, rnq1, sphingolipid, ubiquinone

## Abstract

Within the field of amyloid and prion disease there is a need for a more comprehensive understanding of the fundamentals of disease biology. In order to facilitate the progression treatment and underpin comprehension of toxicity, fundamental understanding of the disruption to normal cellular biochemistry and trafficking is needed. Here, by removing the complex biochemistry of the brain, we have utilised known prion forming strains of *Saccharomyces cerevisiae* carrying different conformational variants of the Rnq1p to obtain Liquid Chromatography-Mass Spectrometry (LC-MS) metabolic profiles and identify key perturbations of prion presence. These studies reveal that prion containing [*RNQ^+^*] cells display a significant reduction in amino acid biosynthesis and distinct perturbations in sphingolipid metabolism, with significant downregulation in metabolites within these pathways. Moreover, that native Rnq1p appears to downregulate ubiquinone biosynthesis pathways within cells, suggesting that Rnq1p may play a lipid/mevalonate-based cytoprotective role as a regulator of ubiquinone production. These findings contribute to the understanding of how prion proteins interact *in vivo* in both their prion and non-prion confirmations and indicate potential targets for the mitigation of these effects. We demonstrate specific sphingolipid centred metabolic disruptions due to prion presence and give insight into a potential cytoprotective role of the native Rnq1 protein. This provides evidence of metabolic similarities between yeast and mammalian cells as a consequence of prion presence and establishes the application of metabolomics as a tool to investigate prion/amyloid-based phenomena.

## Introduction

Protein misfolding disease can be loosely separated into two groups: the non-transmissible amyloid diseases (e.g., Alzheimer’s and Huntington’s) and the transmissible amyloid diseases (e.g., Scrapie and Kuru). The amyloid and prion diseases present the same misfolding mechanism and thus prions can be used to recapitulate the biochemical hallmarks of amyloid disease. Prion (proteinaceous infectious particles, 1) proteins can induce native variants of cellular proteins to adopt alternate, abnormally folded conformations that act as templates for the formation of further misfolded protein. This results in a variety of devastating and fatal neurodegenerative diseases in mammals, with prions being the causal agent of Bovine Spongiform Encephalopathy (BSE) in cattle, Creutzfeldt Jakob disease (CJD) in humans and scrapie in sheep (2, 3).

The ten well-characterised prions of *Saccharomyces cerevisiae* provide a unique vantage point from which to study the biochemistry of prion presence, amyloid formation and their influence on cellular function (2, 3, 4, 5). Of these, the [*RNQ^+^*] prion, plays a pivotal role in heterologous prion appearance, facilitating the *de novo* appearance of prions or other PrD-containing proteins (6, 7). Although required for the *de novo* prion appearance, [*RNQ^+^*] plays no role in prion propagation (8, 9). This role as a facilitator to prion formation places this prion in the initial stages of the complex and downstream process of protein misfolding (8, 9).

The [*RNQ^+^*] prion is consistently found at low levels in wild isolates of *S. cerevisiae* (between 6–26% of tested populations) (10, 11, 12). There are arguments for this ability of Rnq1p to aggregate and/or interact with other Q/N rich proteins is key to Rnq1p’s biological role (11, 13, 14), suggesting that the prion determinant of Rnq1p is maintained within wild populations as a sophisticated “bet-hedging” tool (11, 13). Studies of other yeast prions (such as [*GAR^+^*] and [*SMAUG^+^*]) support this Lamarckian inheritance model (15, 16, 17, 18), hypothesizing that prions are conserved as part of an adaptive strategy, allowing continued population growth in diverse environments via the induction of phenotypic plasticity (19, 20, 21).

A non-essential gene, deletion of the Rnq1p determinant is sufficient to convert a strain from [*RNQ^+^*] to [*rnq^−^*], as is the atypical prion treatment of GdnHCl curing (22, 23). Whilst much is known about the prion form of Rnq1p, the native role of the non-aggregated conformation has remained elusive. Cellular presence of the [*RNQ^+^*] prion does not result in a measurable growth defect and is not toxic *per se*, but overexpression of Rnq1p or a polyQ-containing protein in a [*RNQ^+^*] background causes cell toxicity (24). This toxicity of overexpression is not caused by generalised proteomic stress, but instead is as a result of highly specific mitotic arrest (18). This toxic effect can be elevated via the overexpression of molecular chaperones involved in the propagation of amyloid formation and accentuated by their suppression (24), highlighting the role that intermediates to fibrillation play in the toxic effects seen, bringing into question their interactions with normal cellular functioning.

The use of metabolomics as a tool to investigate amyloidosis and prion formation is not without precedent. Many human blood and plasma studies have focused on biomarker recognition for well-known neurodegenerative disorders (25, 26, 27, 28, 29). The acknowledgment of models outside of the relatively small group of mammals is lacking, even among those who actively encourage multidisciplinary approaches (30). It is argued that the shortfalls of prion or amyloid research that focuses on higher organisms, results from a general lack of understanding regarding the underlying disease biology, highlighting the pivotal role that relatively biochemically simple model organisms, such as *S. cerevisiae* employed here, can have in unravelling such complexities (31).

This work attempts to identify the metabolic pathways that are perturbed due to the presence of the [*RNQ^+^*] prion. Global metabolic comparisons of prion-free [*rnq^−^*] and prion-containing [*RNQ^+^*] *S. cerevisiae* strains aim to identify biomarkers and/or metabolic pathways that are indicative of prion presence/disruption. It is hypothesised that this will aid understanding of amyloid formation and relevant interactions within normal cellular biochemistry, without the restrictions imposed by mammalian cell death. To differentiate the observed metabolic changes caused specifically by [*RNQ^+^*] prion presence from those caused by a general cellular stress response, the study also accounts for perturbations in a prion-free [*rnq^−^*] strain subjected to a mild oxidative stress, thus, ensuring that the observed metabolic changes are specific for prion presence rather than simply indicative of a general cellular stress response. To support this work, metabolomic comparisons between prion-free, prion-containing and a rnq1Δ deleted strain will be used to reveal a possible role for native Rnq1p. In addition, following literature suggestions of stringent consideration of data analysis programmes when conducting global metabolic analysis, two data analysis programmes, MZMine2 and XCMS, were employed for increased rigour and confidence in the analysis (32).

## Results

### Cross comparison of data analysis programmes reveals a startling difference in the number of identified features.

**Figure 1:**
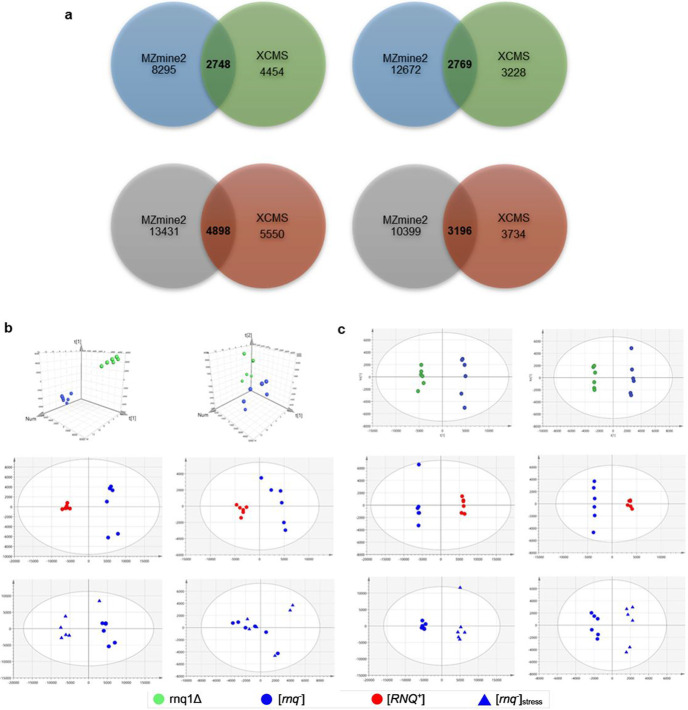
a) Venn diagrams comparing rnq1Δ and [*rnq^−^*] data and [*rnq^−^*], [*RNQ^+^*] and [*rnq^−^*]_stress_ data, respectively, in MZmine2 (blue/grey) and XCMS (green/red). PIM results are shown on the left and NIM results on the right. The overlapping section in the centre depicts the total number of m/z values reported by both analysis programmes in bold. b) PCAs shown depict analysis of knockout (KO, rnq1Δ modelled with [*rnq^−^*]), prion presence (PP, [*RNQ^+^*] modelled with [*rnq^−^*]), and oxidative stress (OS, [*rnq^−^*] modelled with [*rnq^−^*]_stress_ data) (top to bottom). rnq1Δ shown using green circles, [*rnq^−^*] is shown using blue circles, [*RNQ^+^*] is shown using red circles and [*rnq^−^*]_stress_ is shown using blue triangles. PIM results are shown on the left and NIM results on the right. R^2^ and Q^2^ values are as follows PIM - KO, R^2^ = 0.451 Q^2^ = 0.312, PP, R^2^ = 0.712 Q^2^ = 0.523, OS, R^2^ = 0.570 Q^2^ = 0.206 NIM - KO, R^2^ = 0.622, Q^2^ = 0.316, PP, R^2^ = 0.807, Q^2^ = 0.556, OS, R^2^ = 0.843, Q^2^ = 0.160. n=6 per group. c) OPLSDAs shown depict analysis in with the same structure as PCAs. R^2^ and Q^2^ values are as follows PIM - KO, R^2^(X) = 0.544, R^2^(Y) = 0.994, Q^2^ = 0.959, CV-ANOVA *p-value* = 5.85 x 10^−5^, PP, R^2^(X) = 0.748, R^2^(Y) = 0.999, Q^2^ = 0.976, CV-ANOVA *p-value* = 9.26 x 10^−5^, OS, R^2^(X) = 0.649, R^2^(Y) = 0.990, Q^2^ = 0.882, CV-ANOVA *p-value* = 9.45 x 10^−3^. NIM - KO, R^2^(X) = 0.685, R^2^(Y) = 0.998, Q^2^ = 0.953, CV-ANOVA *p-value* = 6.61 x 10^−4^, PP, R^2^(X) = 0.776, R^2^(Y) = 0.997, Q^2^ = 0.936, CV-ANOVA *p-value* = 1.57 x 10^−3^, OS, R^2^(X) = 0.564, R^2^(Y) = 0.977, Q^2^ = 0.743, CV-ANOVA *p-value* = 8.13 x 10^−2^. n=6 per group.

The use of a single data analysis program and the impact that this has on pathway analysis and false discovery rate (FDR) can be considerable. Recent literature suggests careful consideration of the data analysis programs used is capable of controlling for these undesirable variables (32, 33). Results here confirm the startling variance in the number of identified features within MZmine2 and XCMS shown in Fig. 1(a). By utilising a multiprogram comparative analysis of the two most widely used programmes, XCMS and MZmine2, (32) efforts have been made to remove FDRs. Only those that were identified by both programmes were taken as “true features” with those not-overlapping discarded. This approach allowed for increased confidence in subsequent biomarker and pathway investigation. As shown in Fig. 1(a), a total of 2748 positive ionisation mode (PIM) m/z values and 2769 negative ionisation mode (NIM) m/z values were found to be overlapping within the rnq1Δ and [*rnq^−^*] data. Considerably less than within the [*rnq^−^*], [*RNQ^+^*] and [*rnq^−^*]_stress_ data set, with a total of 4898 PIM m/z values and 3196 NIM m/z values.

### Multivariate modelling of comparative metabolomic data reveals clustering and separation between groupings.

To identify the presence of metabolic changes between the test data sets, standard multivariate analysis was employed. Principal component analysis (PCA) using true features as determined by cross comparison demonstrates clear group separation between [*rnq^−^*] and rnq1Δ, [*RNQ^+^*] and [*rnq^−^*]_stress_, respectively, as shown in Fig. 1(b). Overview of the data shows no outliers within the 95% confidence range within each model. The quality of this data was assessed by calculating R^2^ and Q^2^ values shown in Fig. 1(b) these suggest a reasonable fit of the models, in line with typical expectations of biological models. However, the Q^2^ values indicate that residual variation within datasets must exist within the experimental replicates. This variation is visible within the groupings, with PIM results appearing in tighter clusters than NIM supporting the calculated Q^2^ values. Regardless, apparent clustering trends of groups within the models support the progression to OPLS-DA models shown in Fig. 1(c). Here all groupings gave excellent R^2^(X), R^2^(Y) and Q^2^ scores giving confidence in the model’s predictive ability, with all models giving statistically significant CV-ANOVA *p-values* (<0.01).

### Establishing loss of function effects between rnq1Δ, [ rnq ^−^ ] and [ RNQ ^+^ ] reveals considerable overlap in metabolic perturbations between rnq1Δ and [ RNQ ^+^ ], highlighting unique features of native Rnq1p.

**Figure 2:**
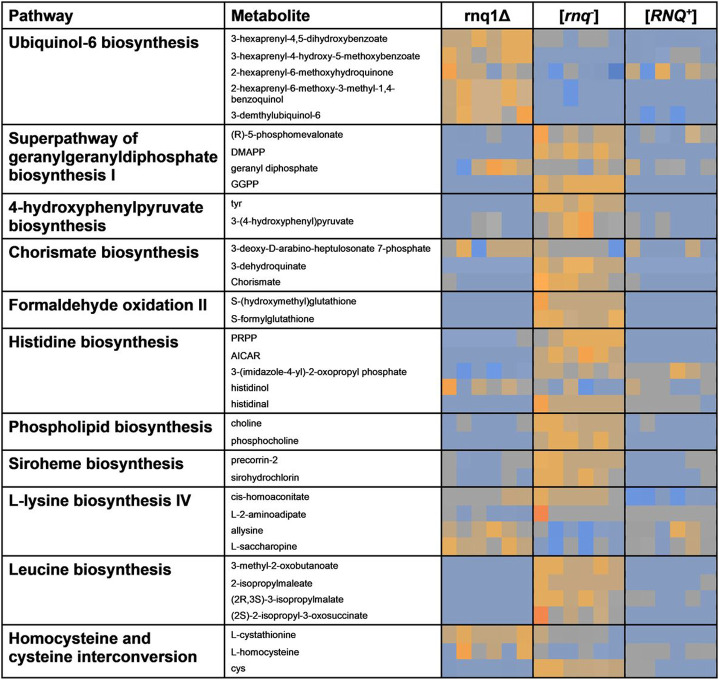
Heat map depicting the most significantly perturbed pathways between rnq1Δ, [*rnq^−^*] and [*RNQ^+^*]. Pathway names and individual metabolites are shown on the left and overlaid data for each data set (n=6) is shown under its respective grouping. Upregulation is shown is dark orange, through to no change in grey and downregulation in dark blue.

To allow for pathway analysis within BioCyc, all significant punitively identified metabolites (as established by Metaboanalyst’s Mummichogg) relative abundances were collated (including both PIM and NIM). Standardised relative abundances of the most significantly altered metabolites (established via Metaboanalyst’s Mummichogg) from the rnq1Δ, [*rnq^−^*] and [*RNQ^+^*] samples (n = 6) were then uploaded to BioCyc’s omics dashboard. The most significantly altered pathways (as determined by DPSS score via BioCyc) are shown in Fig. 2 and reveal a general trend of downregulation of those in cells lacking the Rnq1p in its native confirmation. This study identifies previously unknown perturbed pathways as a result of loss of Rnq1p and in [*RNQ^+^*] prion formation, thus highlighting unique features of native Rnq1p. However, ubiquinol-6 biosynthesis appears significantly downregulated in both [*rnq^−^*] and [*RNQ^+^*] samples suggesting a previously unknown role of Rnq1p as a regulator of ubiquinone-6 biosynthesis. Differences in regulation between loss by deletion of Rnq1p (*rnq1Δ*) and the prion-form of Rnq1p [*RNQ^+^*] were also seen, specifically within the superpathway of geranylgeranyldiphosphate biosynthesis I (via mevalonate) and amino acid synthesis, with the general trend revealing downregulation of these pathways within prion containing cells [*RNQ^+^*] samples when compared to *rnq1Δ*.

### Comparison of [ rnq ^−^ ]_stress_ and [RNQ^+^] cells establishes a generalised stress response in prion containing cells.

**Figure 3:**
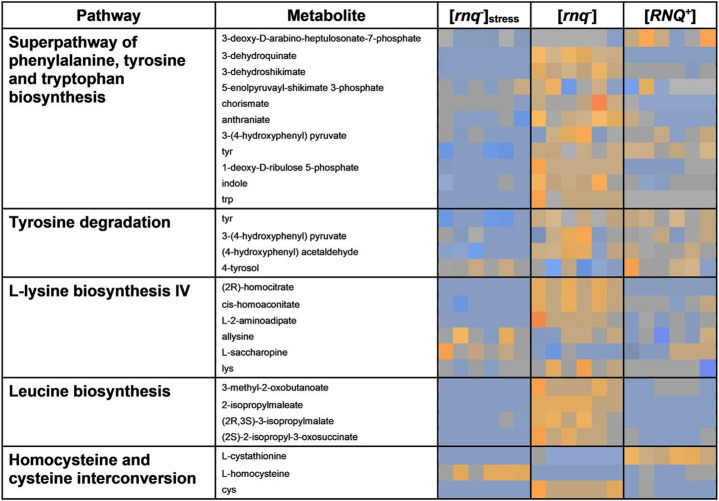
Heat map depicting the most significantly perturbed pathways between [*rnq^−^*] _stress_, [*rnq^−^*] and [*RNQ^+^*]. Pathway names and individual metabolites are shown on the left and overlaid data for each data set (n=6) is shown under its respective grouping. Upregulation is shown is dark orange, through to no change in grey and downregulation in dark blue.

To identify metabolic pathways perturbed by [*RNQ^+^*] prion presence, pathways identified as being specific to loss of Rnq1p native function were removed from the analysis between prion-free ([*rnq^−^*]), prion-containing ([*RNQ^+^*]) and prion-free with mild stress ([*rnq^−^*]_stress_) cells (Fig. 3). The subsequent metabolic pathways identified displayed considerable overlap between [*rnq^−^*]_stress_ and [*RNQ^+^*], confirming the significance of generalised stress response in cells containing prions and justifying its inclusion within this study. Downregulation was seen in metabolites within the L-lysine biosynthesis, leucine biosynthesis and a general trend of downregulation seen in the metabolites within the superpathway of aromatic amino acid biosynthesis.

### Sphingolipids identified as key biomarker/pathways for prion specific metabolic disruption.

**Figure 4:**
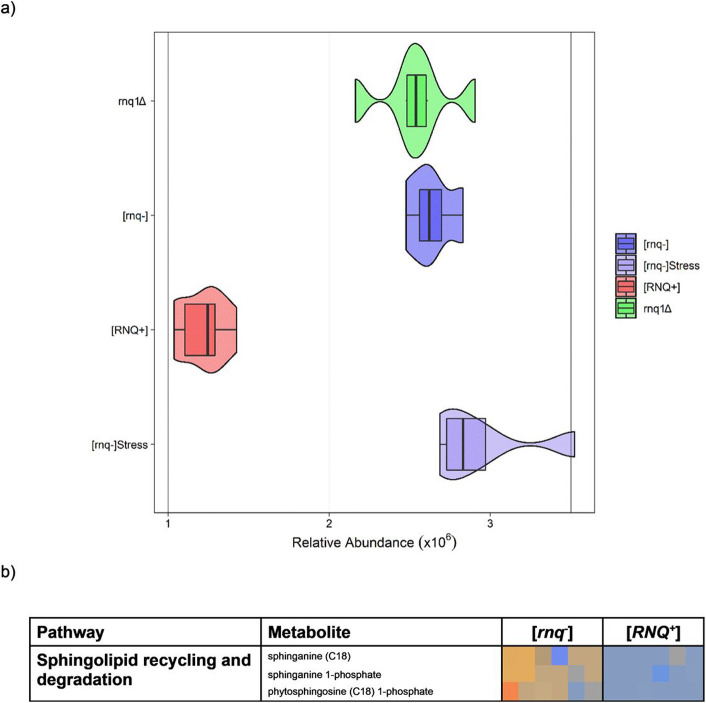
a) Box and whisker plot depicting the change in relative abundance of the metabolite sphinganine with legend shown below indicating sample group by colour. Darker lines in the body of the box depict the mean line and error bars show quartile ranges from the mean. b) Heat map depicting the most significantly perturbed pathway between [*rnq^−^*] and [*RNQ^+^*], following the removal of pathways implicated as being involved in loss of function and overall stress response. Pathway names and individual metabolites are shown on the left and overlaid data for each data set (n=6) is shown under its respective grouping. Upregulation is shown is dark orange, through to no change in grey and downregulation in dark blue.

To identify metabolic changes caused solely by the presence of the [*RNQ^+^*] prion, those perturbed features identified due to loss of Rnq1p function effects and those caused by general stress indicators (as discussed above) were removed from the top 20 most perturbed pathways identified from the prion-free ([*rnq^−^*]) vs prion-containing ([*RNQ^+^*]) analysis (Fig. 4(b)). A single pathway remained that exhibited a significant downregulation in sphingolipid recycling and degradation in prion-containing [*RNQ^+^*] cells, further characterised by a significant reduction in the relative abundance of sphingoanine in cells containing the [*RNQ^+^*] prion (Fig. 4(a)). Sphingoanine is a key metabolite of the early sphingolipid biosynthetic pathway that originates from the combination of palmitoyl CoA and serine and is a precursor to ceramide and is common to both yeast and mammals.

## Discussion

### Using a multiprogram approach provides robust data analysis

The increasing size and complexity of metabolomics experimental data sets (especially untargeted metabolomics) have created a need for faster, more accurate and reliable computational pre-processing. This has led to the availability of variety of programmes used to construct extracted ion chromatograms (EIC), detect and annotate EIC peaks, align samples, identify and relatively quantify analytes (33). With a variety of algorithm processes now underpinning data analysis via any of these methods, concerns have arisen about the capabilities and performance of these programmes relative to each other (32, 33, 34). The large number of different m/z values we obtained from utilising two programmes separately (XCMS and MZmine2) to analyse our own data sets supports these concerns (Fig. 1a). To provide a robustness to our findings, we utilised a cross comparison of overlapping m/z values from both XCMS and MZmine2 (32) thus provide confidence that the resulting perturbations we subsequently report represent real changes that have occurred under the experimental conditions.

### Native Rnq1p Plays A Key Role In Lipid/mevalonate-based Metabolism Regulation

Despite many years of study, the cellular role for native Rnq1p beyond its role in prion generation has remained elusive. Comparison of the metabolic pathways identified from prion-free cells [*rnq^−^*] with those obtained from the rnq1Δ deletion mutant and loss of function of Rnq1p in its amyloid state ([*RNQ^+^*]), reveal an upregulation of those pathways involved in the biosynthesis of many amino acids and key cellular metabolites (Fig. 2), with approximately 70% of the pathways perturbed as a direct result of the loss of function of Rnq1p. Comparison of the perturbations observed between the Δrnq1-deletion mutant and prion-free [*rnq*] establishes that Rnq1p in its native role can reduce the cell’s capacity to synthesise many of these important compounds.

Interestingly, this is not the case within the most significantly perturbed pathway, that of ubiquinol-6 biosynthesis, which is considerably upregulated within cells that do not contain Rnq1p (*rnq1Δ* deletion mutant) compared to those that do contain Rnq1p [*rnq^−^*]. Ubiquinol-6 in its reduced and oxidised forms (ubiquinone), plays a pivotal role within the mitochondrial-based electron transport chain, residing within the inner mitochondrial membrane and acting as a lipid-soluble electron carrier and central electron pool, passing electrons on to complex III of the respiratory chain (35). Ubiquinol-6 pathway metabolites in their reduced forms also act as antioxidants, protecting membrane phospholipids and lipoproteins from lipid peroxidation (36). Rnq1p is known to localise to the cytoplasm (37), not the mitochondria, therefore the observed perturbations in the ubquinol-6 biosynthetic pathway are likely the result of an earlier change. The ubiquinol-6 biosynthesis pathway feeds into hexaprenyl diphosphate biosynthesis, which in turn leads to the superpathway of geranylgeranyldiphosphate biosynthesis I (via mevalonate), terminating at ergosterol biosynthesis. Little is known about the regulation of this pathway (36, 38, 39), although it has been suggested that this regulation is actioned via mevalonate metabolism (40, 41). The directional switch in regulation observed in our data between ubiquinol-6 and the superpathway of geranylgeranyldiphosphate biosynthesis I (via mevalonate; Fig. 2), helps support this notion.

Based on these observations, we suggest that Rnq1p could act as an adaptive regulator of these metabolic pathways under limited stress. Present in wild-populations, (10, 11, 12), the native conformation of Rnq1p in times of limited stress may interrupt/interfere with the regulation of ubiquinol-6 biosynthesis causing a relative downregulation effect. It is proposed that the cellular metabolites and resources are diverted to the production of sterols instead, upregulating the production of more long-term membrane bound cytoprotective lipids. Rnq1p however is intrinsically disordered and so under stress, will readily misfold into its prion form, behaving much like a switch. Conversion to the prion form results in a downregulation of the production of membrane bound cytoprotective lipids. This suggests that Rnq1p plays a lipid/mevalonate based cytoprotective role as an adaptive regulator switch between these cellular defence strategies.

#### [ RNQ ^+^ ] induces widespread metabolomic downregulation including amino acid downregulation

In the absence of Rnq1p due to gene loss, a clear upregulation of the ubiquinol-6 biosynthesis was observed (Fig. 2). However, in prion-containing [*RNQ^+^*] cells where Rnq1p has misfolded from its native conformation to an inactive and aggregated state, a downregulation in ubiquinol-6 biosynthesis and the superpathway of geranylgeranyldiphosphate biosynthesis I (via mevalonate) (Fig. 2) is observed. This is contrary to expectations, as it could be thought that the presence of the [*RNQ^+^*] prion would generate similar “loss of native function” perturbations on metabolism as observed within a rnq1Δ deletion mutant.

A possible explanation for this observation is that prions are known to co-localise around mitochondria within the cell and that possibly this effect is mirroring the downregulation of the ubiquinol-6 pathway seen in [*rnq^−^*] cells of this pathway. However, we also observed that prion containing samples display a widespread downregulation in general biosynthesis, including many pathways that either are or directly affect amino acid regulation. Intriguingly, amino acid restriction in *S. cerevisiae* is beneficial to the overall health of cells, promoting longevity (42, 43, 44), suggesting that maintaining a [*RNQ^+^*] state in wild populations could aid survival by limiting amino acid biosynthesis and instigating amino acid-based dietary restriction. An effect known to have positive effects on longevity, elucidating the findings of increased chronological lifespan in prion containing yeast (45).

### Widespread Metabolomic Downregulation Observed As Part Of A Generalised Metabolic Stress Response

The metabolic perturbations observed within prion-free [*rnq^−^*] cells under normal conditions and those under mild-oxidative stress conditions (Fig. 3) were compared to those found within prion-containing [*RNQ^+^*] cells. Many of the changes were found to overlap, namely the downregulation of notable amino acid biosynthesis pathways and those involved in mitigating mild oxidative stress conditions, indicating that presence of the prion initiated a similar, generalised stress response. Arguably the perturbations observed in response to prion presence was found to be less severely downregulated than those found within the [*rnq^−^*]_stress_ group, suggesting that although yeast prions may not be entirely benign, the cellular damage that occurs in response to them is not overwhelming for cells. However, importantly, differences in perturbations were observed between those caused by mild oxidative stress and those caused by the presence of the prion ([*RNQ^+^*]). These [*RNQ^+^*] prion-only differences were found to be within the superpathway of the phenylalanine, tyrosine and tryptophan biosynthetic pathway. Measurement of metabolites within this pathway offer potential for their use as indicative biomarkers for [*RNQ^+^*] prion presence by permitting the distinction between the cellular response as a result of oxidative stress to that of misfolded protein presence.

Further supporting evidence for the involvement of the tyrosine biosynthetic pathway in protein misfolding and aggregation as identified in this study comes from a separate study on PD (Parkinson’s Disease) were *in vitro* tyrosine residues in patients with PD were found to be sequestered into covalently crosslinked homodimers that appear to play a key role in the formation of seed and oligomeric species (46). Without confirmation of the presence of such a dimer in yeast, it is possible to only speculate that such an event may be occurring within yeast prions too and so enabling the formation of the oligomeric stages that precede amyloid formation.

### Prion-specific Downregulation Of Long Term Cytoprotective Cellular Pathways And Sterols

The most significant perturbation that occurred due to the presence of the [*RNQ^+^*] prion is the down regulation of sphingolipid recycling and degradation (shown in Fig. 4(a + b)) and characterised by downregulated levels of sphingoanine, sphingoanine 1-phosphate and phytosphingoanine 1-phosphate. The sphingolipids, alongside the phospholipids and the sterols, are synthesised mainly within the endoplasmic reticulum (ER) (47) an organelle that plays a key role in the promotion of the correct assembly and folding of newly synthesised proteins. Due to this essential role, monitoring of the ER is crucial to cellular success and when subject to stresses (such as the accumulation of misfolded proteins and perturbations in lipid synthesis) the ER is acted heavily upon by the unfolded protein response or UPR, which aims to assist protein folding, remove misfolded proteins, and promote lipid synthesis (48). During yeast daughter growth, ER stresses are known to initiate a “diffusion barrier response”, which prevents damaged proteins and metabolites from entering daughter cells during budding (49). Experiments on Hsp70p revealed that this barrier prevents misfolded proteins from entering daughter cells and confirm that the sphingolipids play a key and vital role in this compartmentalization and barrier formation (49). Posited to form the bottom part of a fatty molecules layer, the sphingolipids, are thought to act as a skeleton to allow this diffusion barrier to form (49). Given that the mechanism which prions use to proliferate from mother to daughter cells is now understood to involve ‘an unknown’ mechanism of partitioning (50), it seems reasonable, based on the observations in sphingolipid degradation, to speculate that these diffusion barrier forming sphingolipids may have a critical role within this partitioning between mother to daughter cells.

Alternatively, or additionally, it is possible that the rate of degradation of sphingolipids is lower in prion containing [*RNQ^+^*] samples simply due to the cells using all their sphingolipid resources available to cope with the number of misfolded proteins. Indeed, within amyloid biology it is well documented that microdomains are formed by the sphingolipids and cholesterols that act as sites for the binding and oligomerisation of amyloidogenic proteins (51). In cells carrying the mammalian prion (PrP^SC^), sphingolipid rafts present in cell plasma membranes have been found to decrease in concentration (52). It is proposed that sphingolipid rafts are used by cells to aid in the formation of the PrP^SC^ from its normal conformer PrP^C^ (52). Thus, it could be hypothesised that the observed downregulation of sphingolipids observed in this study as a result of [*RNQ^+^*] presence may be as a result of continued use or containment of sphingolipid rafts within the process of misfolding and subsequent aggregation of [*RNQ^+^*]. Despite the common inference that connects the mechanistic actions of mammalian prions/amyloids with the yeast prions, this has not previously been observed in yeast and provides evidence of homology between yeast prions and mammalian prions and amyloids.

## Conclusion

To reveal the metabolic consequences on the cell caused by prion presence, we undertook a LC-MS metabolomics study to identify metabolic perturbations caused by native Rnq1p protein loss, general stress response un-related to prion presence, and prion presence. We utilised the Rnq1p status of cells to define prion absence [*rnq^−^*] or presence [*RNQ^+^*]; choosing to study [*RNQ^+^*] due to its presence in wild yeast stains and its hierarchal role in the induction of other yeast prions such as [*PSI^+^*]. By identifying and removing the metabolic perturbations caused by loss of Rnq1p function and those caused by a general stress response, we have revealed the consequences of prion presence on yeast global cell metabolism, namely the downregulation of the sphingolipid biosynthesis and recycling pathway. Previously identified in non-metabolomic mammalian prion studies, the involvement of sphingolipid metabolism in yeast prion biology helps to cement the homology between yeast and mammalian systems, supporting the use of this valuable model. In addition, we establish a potential role for native Rnq1p as a responsive switch between short term and long term cytoprotection, reinforcing the hypothesis that the native role of Rnq1p has a significant link to its prion-only role, one which extends beyond cause and effect. Although further work is needed to explore the mechanism of this role. Indeed, this type of intrinsically disordered protein fulfilling independent roles in different conformations, provides a causal mechanism for the phenoplasticity which is commonly observed (19, 20, 21). Mammalian cells do not possess a directly homologous protein to Rnq1p, but this study may help direct the search for an intrinsically disordered protein that performs an analogous role. If such a protein exists, targeted disruption/upregulation/downregulation of the pathways identified here may mitigate effects in mammalian cells too.

## Materials And Methods

### Strain and cultivation conditions

The *S. cerevisiae* strain used in this study were derivatives of 74-D694 (*MATa ade1-14(UGA) trp1-289(UAG) ura3-52 his3-Δ200 leu2-3, 112*). Yeast harbouring [*RNQ^+^*] and knockout strain Δrnq were kind gifts from the Kent Fungal Group. Yeast were grown at 30°C with shaking at 180 rpm in synthetic complete (SC) media (2% (w/v) glucose, 0.17% Yeast Nitrogen Base (without amino acids, without ammonium sulphate), 0.5% ammonium sulphate, the appropriate concentration of yeast synthetic complete supplement mixture or synthetic complete drop-out media supplement). Transient growth on SC media containing 3mM guanidine hydrochloride (GdnHCl) was used as a curing agent in the media of *S. cerevisiae* cells that required a [*prion^−^*] status. Mild oxidative stresses were achieved by the addition of H_2_O_2_ (final concentration 0.2 mM) to the appropriate culture mediums. Cultures were grown using the filter culture method, (as described by 53, 54, 55).

### Metabolite sample preparation

Analytical grade standards were supplied by Sigma Aldrich. Quenching was achieved by adaption of cold methanol protocol (56), via submersion of entire filter membrane. Metabolite extraction was performed on the resultant cell pellets using the boiling ethanol technique (57). Briefly, each tube was taken from the − 80°C and 5 ml 75% (v/v) boiling ethanol was added (pre-heated). Each tube was immediately vortexed and placed in a water bath at 80°C. After 5 min each tube was cooled on ice for 3 min, followed by centrifugation (5000xg, 5 minutes, −20°C, precooled). Extracts were then stored at −80°C until further use. Immediately prior to mass spectrometry experimentation all extracts were concentrated by speed vacuum at 35° C for ≈ 3 hours. Following resuspension in 500μL of LC/MS grade water samples were lyophilised overnight. Lyophilised samples were then resuspended in 200μL of 0.1M formic acid, vortexed and loaded into vials.

### UHPLC-QToF-MS/MS experiments

Metabolites were separated using a Waters Acquity UHPLC system coupled to an ACQUITY SYNAPT G2-Si Mass Spectrometer operating in electrospray resolution mode (Waters Corporation, Wilmslow, UK). Reversed phase (RP) separation was employed using a 1.7 μm C18 BEH column (Waters Corporation, Wilmslow, UK), heated to 35°C and operated with a 10-minute gradient from 0–50% acetonitrile (0.1% formic acid) at a flow rate of at 500 nL/min. Detection was carried out in both positive and negative mode, respectively. A capillary voltage of 2.5 kV and a cone voltage of 40 V were used. Nitrogen, the desolvation gas, was set to 800 L/hr at a temperature of 400°C. MSE continuum mode was used for data acquisition over the mass range of 50-1200 m/z, with a scan time of 1 second, which was programmed to step between low (10 eV) and elevated ramp collision energies (20–35 eV) with argon was used as the collision gas. The data was collected in Data Independent Acquisition (DIA) MS/MS mode. Leucine-enkephalin (556.2771 m/z) was used as a lock-spray standard injected every 10 seconds for 1 second. Water blanks were also analysed every 50 samples to check for contamination. For quality control, 25 μL of each sample was pooled run ≈ every 10 samples for quality control.

### Data and pathway analysis

The raw data (.raw) files were automatic peak detected via Masslynx (Waters, Version 4.1, Waters, Milford, MA, USA) and then converted to .mzML format using Proteowizards MSConvert. To ensure robustness, the subsequent mzML. files were then analysed using two separate data analysis programmes, MZmine2 and XCMS (parameters available in supplementary materials), and a comparative analysis using overlapping features of the two programmes using VBA programming, adapted from Li *et al.* (2019). Statistical analysis was performed using SIMCA 14.1 (Umetrics). Punitive compounds and pathways were identified using Metaboanalyst’s Mummichogg function MS Peaks to Pathways (58). BioCyc’s omics dashboard was used to compare relative abundances of the most significantly altered metabolites. BioCycs cellular overview was then used, with the same relative abundance data, to create pathway tables and pathway collages of the most significantly altered metabolic pathways, ranked via DPPS score (59). These pathways were then overlaid with standardized z-score data for visualisation.

## Data Availability

The data obtained in this study will be accessible at the NIH Common Fund's NMDR (supported by NIH grant, U01-DK097430) website, the Metabolomics Workbench, https://www.metabolomicsworkbench.org. Once the data has been assigned a project ID this will be made available. Until this time the data is available via the link below https://drive.google.com/file/d/1o1gQklpNtAL0OIn0n0xKVfZFGxWH3N92/view?usp=sharing
